# A Combination Prediction Model of Long-Term Ionospheric foF2 Based on Entropy Weight Method

**DOI:** 10.3390/e22040442

**Published:** 2020-04-14

**Authors:** Hongmei Bai, Feng Feng, Jian Wang, Taosuo Wu

**Affiliations:** 1 School of Microelectronics, Tianjin University, Tianjin 300072, China; wulanfree@163.com (H.B.); 1016204014@tju.edu.cn (J.W.); wutaosuo@126.com (T.W.); 2 School of Mathematics and Statistics, Hulunbuir College, Hulunbuir 021008, China; 3 Tianjin Key Laboratory of Imaging and Sensing Microelectronic Technology, Tianjin University, Tianjin 300072, China; 4 Department of Electronics, Carleton University, Ottawa, ON K1S 5B6, Canada; 5 Qingdao Institute for Ocean Technology, Tianjin University, Qingdao 266237, China

**Keywords:** ionosphere, foF2, entropy weight method, combination prediction model

## Abstract

It is critically meaningful to accurately predict the ionospheric F2 layer critical frequency (foF2), which greatly limits the efficiency of communications, radar, and navigation systems. This paper introduced the entropy weight method to develop the combination prediction model (CPM) for long-term foF2 at Darwin (12.4° S, 131.5° E) in Australia. The weight coefficient of each individual model in the CPM is determined by using the entropy weight method after completing the simulation of the individual model in the calibration period. We analyzed two sets of data to validate the method used in this study: One set is from 2000 and 2009, which are included in the calibration period (1998–2016), and the other set is outside the calibration cycle (from 1997 and 2017). To examine the performance, the root mean square error (RMSE) of the observed monthly median foF2 value, the proposed CPM, the Union Radio Scientifique Internationale (URSI), and the International Radio Consultative Committee (CCIR) are compared. The yearly RMSE average values calculated from CPM were less than those calculated from URSI and CCIR in 1997, 2000, 2009, and 2017. In 2000 and 2009, the average percentage improvement between CPM and URSI is 9.01%, and the average percentage improvement between CPM and CCIR is 13.04%. Beyond the calibration period, the average percentage improvement between CPM and URSI is 13.2%, and the average percentage improvement between CPM and CCIR is 12.6%. The prediction results demonstrated that the proposed CPM has higher precision of prediction and stability than that of the URSI and CCIR, both within the calibration period and outside the calibration period.

## 1. Introduction

The critical frequency of the F2 layer of the ionosphere (foF2) is one of the most important parameters in various civil and military applications [1,2]. The foF2 parameter is not only used for designating the maximum usable frequency (MUF) and the lowest usable frequency (LUF) of the high frequency (HF) communication systems [3], but also its spatiotemporal variability will affect the efficiency of radar and navigation systems [4,5]. Therefore, the improvement of existing foF2 prediction models will play an important role in the planning and frequency management of HF radio systems, HF automatic link establishment, and global positioning satellites. 

Popular methods used to predict the foF2 include the International Reference Ionosphere (IRI) [6,7,8,9,10,11,12], Artificial Neural Network (ANN) model [13,14,15,16,17,18,19], Support Vector Machine technique (SVM) [20,21], and so on. The most widely used global ionospheric model for the prediction of ionospheric parameters (such as foF2) is called the IRI, and is the internationally recognized model for predicting ionospheric parameters [3,4,5,6,7,8,9,10,11]. As more and more new ground and space data sets of the ionosphere become available, the IRI Working Group (the Committee on Space Research (COSPAR) and the URSI) continue to develop and improve existing IRI models. Some important versions of the IRI model have been released including the IRI-78 [6], IRI-1990 [7], IRI-2000 [8], IRI-2007 [9], IRI-2012 [10], and IRI-2016 [11]. Therefore, researchers compared each newly developed ionospheric parameter model with the IRI model to ensure that the new method has better performance than the IRI model [1]. The IRI-2016 has been used in this study.

The IRI model provides two options, which are International Radio Consultative Committee (CCIR) [22] and URSI [23], respectively. The reliability of the IRI model is affected by the coverage of the observation network [24,25,26]. The IRI model performs better in the northern hemisphere than in the southern hemisphere because that is the region with the highest density of ionosondes stations [16]. We also noticed that the foF2 observation data was compared with the foF2 prediction data of the IRI model (including URSI and CCIR coefficients) in literatures [4,27,28,29,30,31]. These studies have revealed that the foF2 data predicted by both URSI and CCIR deviate (overestimated or underestimated) from the observation data of the foF2 on many occasions, especially at low latitude stations. Bai et al. compared the observed value with the predicted value of the IRI model at Darwin (12.4° S, 131.5° E) in Australia [4,32]. The comparison results show that the predicted value of the IRI model needs to be further improved. 

In this paper, we propose to systematically combine the two prediction models (URSI and CCIR) with the aim of establishing a more accurate long-term foF2 prediction model at Darwin. The entropy weight method was used to determine the weighting parameter for each individual prediction model (URSI or CCIR) in the proposed combination prediction model (CPM) according to variability of prediction error sequences. It is known that the combined forecasting model, established by the entropy weight method, has been widely used in various fields, such as environmental management [33], energy [34], and software reliability [35]; mainly because the method is simple to calculate, easy to grasp, strong in stability, and good in the computing accuracy. The results of this model were compared with the URSI and CCIR models for predicting the behavior of the foF2.

## 2. Data and Methodology 

### 2.1. Database

As an important ionospheric parameter, the foF2 is commonly used in radio wave propagation [4,5]. The monthly median values of the ionospheric parameters foF2 obtained at the ionosonde station of Darwin are used in this paper. We selected the observed data during the interval of 1997–2017 to develop the proposed CPM. The measured data were downloaded in two parts. The data covering the years of 1997–2013 were downloaded from the Australian Government Bureau of Meteorology Space Weather Services (http://www.sws.bom.gov.au), and the data covering the years of 2014–2017 were downloaded from the National Oceanic and Atmospheric Administration (NOAA) (ftp://ftp.swpc.noaa.gov/pub/lists/iono_month). The collected data for the monthly median values of the foF2 covers at least 21 days (24 h for each day) per month to assure the reliability [4,30]. In addition, the IRI model is available in the FORTRAN source code at http://irimodel.org. 

### 2.2. Methodology

Entropy is introduced into the information theory by Shannon [36]. The entropy weight method was used to determine the weight coefficient of different individual predicting models based on the variability of prediction error sequences (such as the relative error) [33,37,38,39]. If the information entropy value is small, it means that the data are provided by numerous useful attributes, the weight assigned to the evaluation object should be larger and vice versa [33,37,38,39,40]. 

Suppose X is a random variable, $P_{X}(x)$ is a probability density function, and the Shannon entropy, H(X), is formulated as [41]:

(1) $$H(X)=-\sum_{x\in X}p_{X}(x)\ln p_{X}(x),$$

where x represents a value of the X. 

Using entropy weight method to evaluate weighting coefficients of the monthly median foF2 prediction, the specific steps are as follows:

(1) The weight of $e_{ij}$ in the *j*th year of the *i*th individual model can be calculated as:

(2) $$P_{ij}=\frac{\left|e_{ij}\right|}{\sum_{j=1}^{n}e_{ij}}(i=1,2),$$

where $e_{ij}$ is defined as the average relative error between the observed monthly median foF2 value and the prediction monthly median foF2 value of the *i*th individual model for the *j*th year.

(2) Calculate the entropy of the *i*th individual model:

(3) $$H_{i}=-\frac{1}{\ln n}\sum_{j=1}^{n}p_{ij}\ln p_{ij}$$

If $p_{ij}=0$, then $p_{ij}\ln p_{ij}=0$.

(3) Calculate the weight of *i*th individual model:

(4) $$w_{i}=\frac{1-H_{i}}{m-\sum_{i=1}^{m}H_{i}}\;(i=1,2,m=2),$$

where $0\leq w_{i}\leq 1,\sum_{i=1}^{m}w_{i}=1$.

(4) Calculate the monthly median value of the foF2 predicted by the CPM:

(5) $$Y=\sum_{i=1}^{2}w_{i}y_{i},$$

where $y_{i}$ represents the prediction value of the monthly median foF2 value of the *i*th individual model.

The root mean square error (RMSE) is used to measure the deviation between the predicted value of the CPM, URSI, and CCIR, and observed values. The RMSE is formulated as:

(6) $$RMSE=\sqrt{\frac{1}{N}\sum_{i=1}^{N}{\left(foF2_{obs}-foF2_{pred}\right)}^{2}}$$

where *N* denotes the total number of data sets, $foF2_{obs}$ represents the observed monthly median foF2 value, and $foF2_{pred}$ represents the corresponding predicted monthly median foF2 values of the CPM, URSI, and CCIR.

In addition, the improvement percentage of foF2 (foF2_IMP_[%]) was also used as an evaluation indicator for comparing the predicted results of CPM and IRI model, as defined in:

(7) $$foF2_{IMP}(\%)=\frac{RMSE_{IRI}-RMSE_{CPM}}{RMSE_{IRI}}\times 100\%,$$

where RMSE_IRI_ represents the RMSE for the IRI model (URSI or CCIR options), and RMSE_CPM_ represents the RMSE for the proposed CPM.

## 3. Results and Discussions

The calibration data are selected from 1998 to 2016. Two sets of data were used to validate the prediction performance of the proposed CPM model: (1) the data in the calibration period, namely, 2000 and 2009, (2) the data outside the calibration period, namely, 1997 and 2017.

Firstly, the foF2 prediction results of the URSI and CCIR were simulated during the calibration period. Secondly, the average relative error between the observed monthly median foF2 value and the prediction monthly median foF2 value of URSI and CCIR was calculated. Finally, Equations (2)–(5), mentioned in Section 2.2, were used to determine the weight coefficients of each individual prediction model in the CPM. The weights of the URSI and CCIR were calculated to be $w_{1}=0.635$ and $w_{2}=0.365$, respectively.

Substituting the weight coefficient into Equation (5), the predicted response of CPM can be obtained from:

(8) $$foF2_{CPM}=0.635\times foF2_{URSI}+0.365\times foF2_{CCIR},$$

where $foF2_{URSI}$ represents the predicted monthly median foF2 value of the URSI, $foF2_{CCIR}$ represents the predicted monthly median foF2 value of the CCIR, and $foF2_{CPM}$ represents the predicted monthly median foF2 value of the CPM.

The results of Equation (8) show that if the actual observed data fall between the predicted data of two individual models, the accuracy of the combined prediction is better than that of each individual prediction method. In other words, if the actual observed data is larger (or smaller) than the predicted data of the two individual models in the combined model, the prediction performance of the combined prediction model may not be as good as that of the individual prediction model.

### 3.1. The Performance of Different Prediction Models within the Calibration Period 

**Figure 1:**
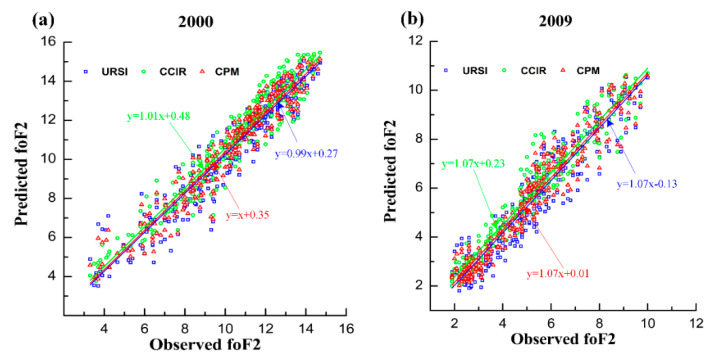
The scatter diagram of the foF2 observation and the foF2 prediction based on the URSI, CCIR, and CPM in 2000 (**a**) and 2009 (**b**) within the calibration period. The linear regression fit for each dataset is shown, together with the equation of this regression line.

In order to present the performance in the calibration period, the scatter diagrams between the monthly median foF2 observation data and the monthly median foF2 prediction data based on the URSI, CCIR, and CPM were plotted. For brevity, Figure 1 shows only the results for 2000 and 2009, which are included in the calibration period. Figure 1 also shows the linear regression fit line for each data set and the equations for that regression line. The X axis represents the foF2 observation data and the Y axis represents the foF2 prediction data for the URSI, CCIR, and CPM. The slope values (equal to 1 or close to 1) of the fitted linear regression of the three prediction models (URSI, CCIR, and CPM) indicate that all three models have a good fitting ability in 2000 and 2009. In Figure 1a, it was also found that most of the scattered points were concentrated at the end of the regression line fitting line, because 2000 was a high solar activity year; that is, the value of the foF2 is relatively large. In Figure 1b, it was also found that most of the scattered points were concentrated at the beginning of the regression fitting line, because 2009 was a low solar activity year; that is, the value of the foF2 is relatively small.

**Figure 2:**
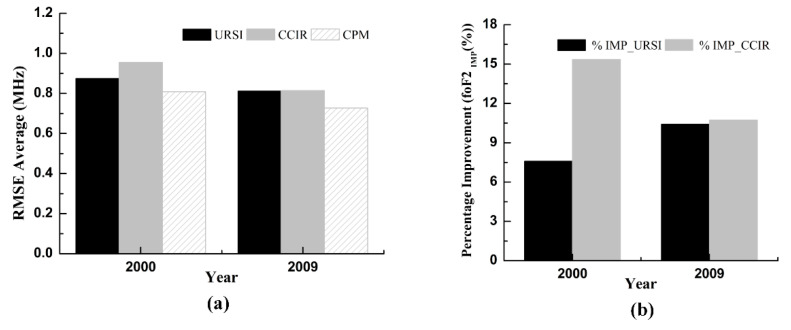
Bar charts showing the yearly performance comparison of the URSI, CCIR and CPM in 2000 and 2009 within the calibration period. (**a**) The root mean square error, and (**b**) the percentage improvement of the CPM over both CCIR and URSI.

The root mean square error (RMSE) and the percentage improvement of the foF2 (foF2_IMP_(%)) were used to examine the yearly performance of the proposed CPM and individual models (CCIR and URSI) for the foF2 prediction in 2000 and 2009. The RMSE average values of the CPM, URSI, and CCIR for the years of 2000 and 2009 are shown in Figure 2a. It can be obviously observed from Figure 2a, that the RMSE average values calculated from CPM are less than those calculated from URSI and CCIR in 2000 and 2009. This shows that the CPM has good predictive performance and stability compared to URSI and CCIR. Figure 2b shows the bar graphs representing the percentage improvement of the CPM over both CCIR and URSI. In 2000, the percentage improvement of the CPM over URSI was 7.59%, and the percentage improvement of the CPM over CCIR was 15.35%. In 2009, the percentage improvement of the CPM over URSI was 10.42%, and the percentage improvement of the CPM over CCIR is 10.73%. It was seen in Figure 2b, that the accuracy of the URSI and CCIR was improved by the proposed CPM.

During the calibration period, the results of the proposed CPM not only has higher prediction accuracy than both URSI and CCIR, but also the highest stability.

### 3.2. The Performance of Different Prediction Models outside the Calibration Period 

**Figure 3:**
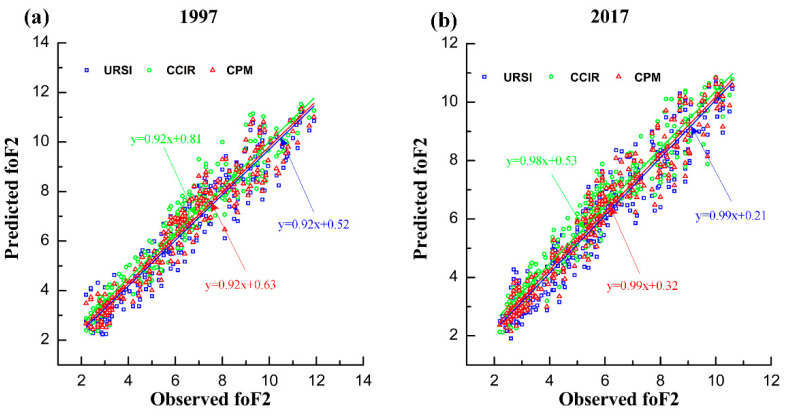
The scatter diagram of the foF2 observation and foF2 prediction based on the three prediction models in 1997 (**a**) and 2017 (**b**) outside the calibration period.

In order to further test the predictive ability of the proposed CPM outside the calibration period (before and after), the data of 1997 and 2017 are used. The scatter diagrams of the foF2 prediction results (URSI, CCIR and CPM) and the foF2 observation data are plotted in Figure 3 (similar to Figure 1). Figure 3 shows that the slope of fitting linear regression of the three prediction models (URSI, CCIR, and CPM) are close to 1 in 1977 and 2017. This indicates that all three models still have a good fitting ability in the 1977 and 2017.

In Figure 3a,b, it can also be found that most of the scattered points were concentrated at the beginning of the regression fitting line, because 1997 and 2017 were low solar activity years; that is, the value of the foF2 is relatively small.

**Figure 4:**
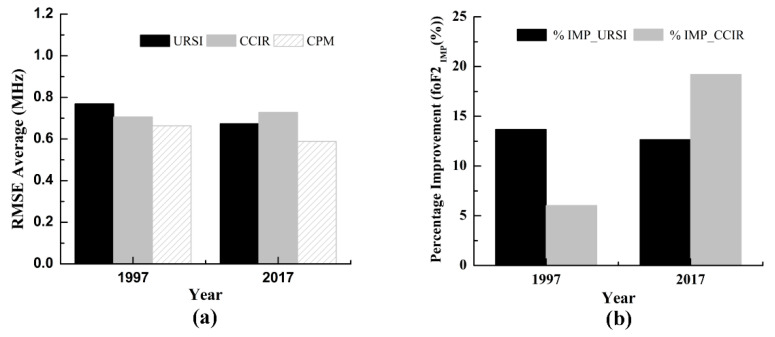
Bar charts showing the performance comparison of the URSI, CCIR, and CPM during the data before (1997) and after (2017) the calibration period. (**a**) The root mean square error, and (**b**) the percentage improvement of the CPM over both CCIR and URSI.

The RMSE and the foF2_IMP_(%) were also used to test the predicting capability of the proposed CPM and each existing model (CCIR and URSI) for foF2 prediction outside the calibration period. The RMSE differences for both years, 1997 and 2017, are shown in Figure 4a. Comparing the RMSE of the CCIR and URSI, it was found that CCIR predicted better performance than URSI in 1997, whereas the results in 2000 were reversed. This indicates that URSI and CCIR do not have good stability. Figure 4a also illustrates that the values of RMSE of the CPM are lower than both the URSI and CCIR models in both 1997 and 2017. These results demonstrate that the foF2 prediction using CPM has better stability than those using URSI and CCIR.

Figure 4b shows that the accuracy of the URSI and CCIR models can be improved by using the proposed CPM. In 2007, the CPM improved the accuracy from the URSI and CCIR models by 13.68% and 6.03%, respectively. In 2017, the CPM improved the accuracy from the URSI and CCIR models by 12.65% and 19.21%, respectively.

Compared with the results obtained from the three models (CPM, URSI, and CCIR) outside the calibration period, the proposed CPM also has the best performance and stronger stability.

## 4. Conclusions

This paper proposed a combined prediction model of the ionospheric foF2 based on the entropy weight method. According to the prediction performance of different individual prediction models, we can determine the weight parameters for each individual prediction model. The monthly median values of the measured foF2 collected from Darwin station were used to develop the proposed CPM. The period of 1998–2016 was selected as the calibration period. The predictive performance of the proposed model was validated using two sets of data, namely, data included in the calibration period (2000 and 2009) and data outside the calibration period (1997 and 2017). The scatter plots between the observed values of the foF2 and the predicted values of the foF2 from three different models were plotted in different years. The scatter plot (Figure 1 and Figure 3) revealed that all three models had good fitting ability. As clearly seen on the yearly RMSE averages (Figure 2 and Figure 4), the RMSE averages obtained from CPM are lower than those obtained from URSI and CCIR. The prediction result shows that, compared with individual models (URSI and CCIR models), the proposed CPM improves prediction accuracy and stability, thereby reducing the overall uncertainty of the prediction. In addition, developing a foF2 prediction model with different coefficients for other locations using the combination prediction model will be the subject of our future research.
